# Tissue Specific Expression of Cre in Rat Tyrosine Hydroxylase and Dopamine Active Transporter-Positive Neurons

**DOI:** 10.1371/journal.pone.0149379

**Published:** 2016-02-17

**Authors:** Zhenyi Liu, Andrew Brown, Dan Fisher, Yumei Wu, Joe Warren, Xiaoxia Cui

**Affiliations:** SAGE Labs, A Horizon Discovery Group Company, Saint Louis, MO, United States of America; Emory University, UNITED STATES

## Abstract

The rat is a preferred model system over the mouse for neurological studies, and cell type-specific Cre expression in the rat enables precise ablation of gene function in neurons of interest, which is especially valuable for neurodegenerative disease modeling and optogenetics. Yet, few such Cre rats are available. Here we report the characterization of two Cre rats, tyrosine hydroxylase (TH)-Cre and dopamine active transporter (DAT or Slc6a3)-Cre, by using a combination of immunohistochemistry (IHC) and mRNA fluorescence *in situ* hybridization (FISH) as well as a fluorescent reporter for Cre activity. We detected Cre expression in expected neurons in both Cre lines. Interestingly, we also found that in Th-Cre rats, but not DAT-Cre rats, Cre is expressed in female germ cells, allowing germline excision of the floxed allele and hence the generation of whole-body knockout rats. In summary, our data demonstrate that targeted integration of Cre cassette lead to faithful recapitulation of expression pattern of the endogenous promoter, and mRNA FISH, in addition to IHC, is an effective method for the analysis of the spatiotemporal gene expression patterns in the rat brain, alleviating the dependence on high quality antibodies that are often not available against rat proteins. The Th-Cre and the DAT-Cre rat lines express Cre in selective subsets of dopaminergic neurons and should be particularly useful for researches on Parkinson’s disease.

## Introduction

In the post-genome era, the ability to control gene expression in a spatial as well as temporal manner is imperative to functional genomics studies and ultimately drug development. The creation of conditional loss/gain-of-functional models is necessary when changes are detrimental to early development. Conditional knockout is generally achieved by employing the Cre-LoxP system [1, 2], which relies on two lines of genetically modified animals: a floxed line in which two 34-base-pair-long LoxP sites are inserted into the target gene of interested, flanking a region critical to gene function, and a Cre line that expresses phage recombinase Cre from a promoter that provides spatial or temporal control. In animals with both the Cre and floxed alleles, the sequence between loxP sites is excised in Cre expressing cells, leading to loss of function of the target gene [2, 3]. In other words, the fidelity of Cre expression determines tissue/cell type specificity of targeted gene disruption. Most available rat Cre lines were generated via random transgenes, with the caveats of possible positional effects as well as incomplete inclusion of regulatory elements of a promoter, and are often only partially recapitulate the desired expression patterns. On the other hand, targeted integration of the Cre coding sequence into an endogenous locus leads to expression of Cre from the genomic context of the target gene, potentially with the same pattern.

The mouse became the dominant model organism for biomedical research largely for being the only species with established pluripotent embryonic stem (ES) cell lines that are genetically malleable [4–6], until the recent development of programmable nuclease technologies, including zinc finger nucleases (ZFNs) [7, 8], transcription activator-like effector nuclease (TALENs) [9, 10] and clustered regularly inter-spaced short palindromic repeats (CRISPRs)-based nucleases [11, 12](For review, see [13, 14]), all designed to introduce double strand breaks at specific DNA sequences and stimulate gene editing.

The rat has many advantages over the mouse for studies related to cognitive neuroscience, cardiovascular diseases, breast cancer, diabetes and drug metabolism [15]. Although successful genetic manipulation of ES cells and the creation of germ line knockout rats have been reported recently [16–18], such procedures are still technically demanding [19]. Instead, microinjection of designer nucleases directly into fertilized eggs to manipulate the genome revolutionized the field of gene editing. As many other species, the rat can now be used in research where it is most biologically relevant.

We recently reported the creation of the first floxed alleles in the rat and along with a Th-Cre rat with an IRES-Cre cassette inserted to the endogenous Th locus using ZFNs [20]. Here we characterized the Cre expression pattern in details and using the same methodologies, created and characterized another Cre line with more limited expression in dopaminergic neurons, DAT-Cre. By using immunohistochemistry and/or RNA fluorescent *in situ* hybridization, we demonstrated that in both Cre lines, the Cre expression pattern in general faithfully recapitulates that of respective endogenous target. And functionally, Cre recombinase mediates efficient excision of floxed alleles in expected cell populations. Interestingly, Cre-mediated excision of the floxed alleles was observed in the maternal germ cells of the Th-cre rats, which could be employed to conveniently create germline knockout from floxed rats. We believe these Cre lines are valuable tools for neurological research.

## Results

### The insertion of the IRES-Cre cassette does not alter the expression pattern of the Th gene in the brain

**Fig 1:**
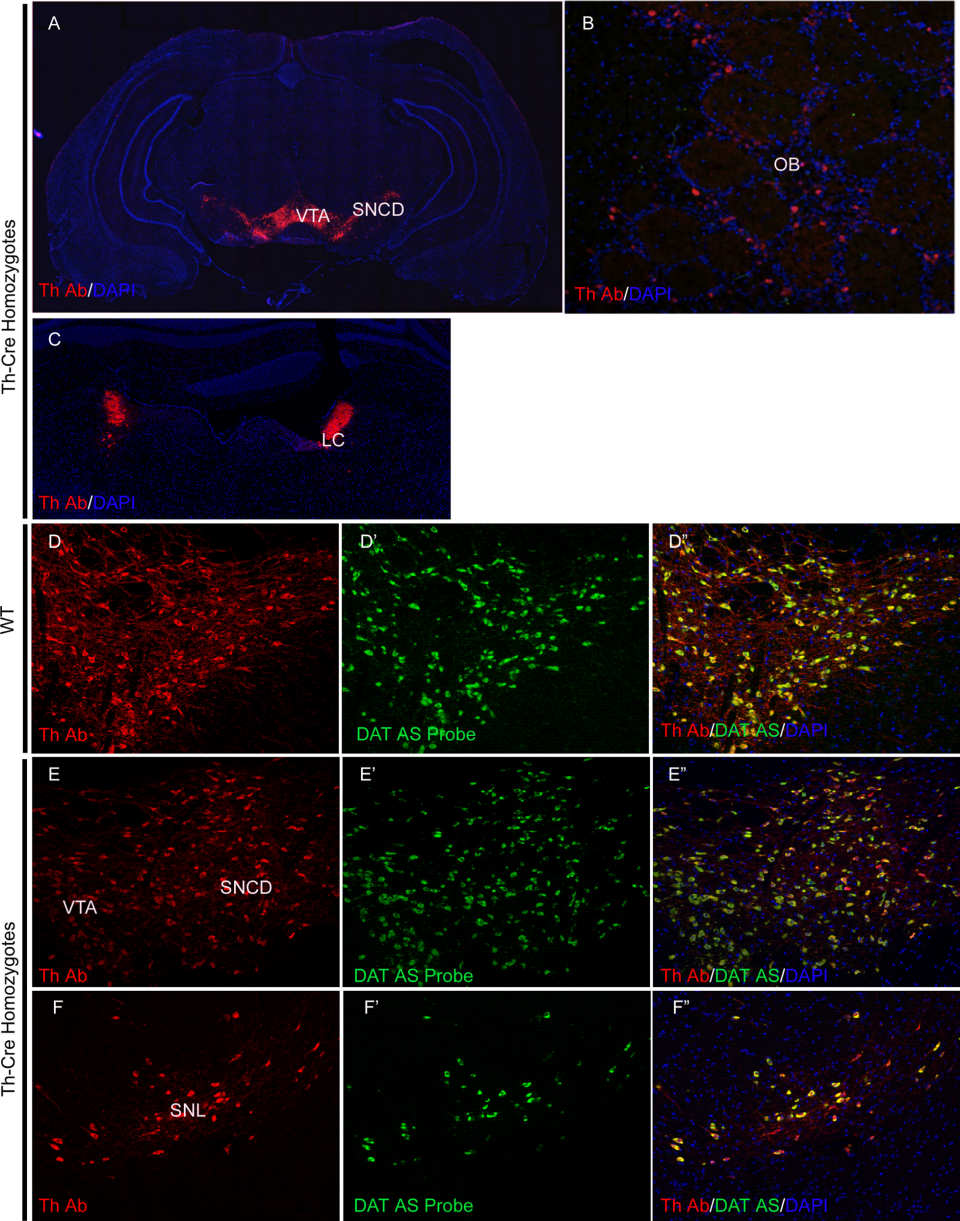
The insertion of the IRES-Cre cassette into the Th locus does not change its expression pattern in the brain. (A-C) Anti-Th antibody staining in the brain of adult homozygous Th-Cre rats; (D-F”), anti-Th antibody (D, E, F) and DAT anti-sense probe *in situ* hybridization (D’, E’, F’) in the brains of wild type adult rats (D and D’) and homozygous Th-cre rats (E, E’, F and F’). AS, anti-sense; LC, locus coeruleus; OB, olfactory bulb; SNCD, substantia nigra compacta dorsal tier; SNL, the lateral part of substantia nigra; SNR, the reticular part of substantia nigra; VTA, ventral tegmental area.

In Th-Cre rat, the IRES-Cre cassette is inserted immediately after the stop codon of the *Th* gene [20]. To examine whether this insertion altered the expression pattern of *Th*, we performed immunohistochemistry with anti-Th antibody on brain sections from homozygous Th-Cre rats. We found that like in the wild type animals, *Th* was strongly expressed in dopaminergic neurons of ventral tegmental area (VTA) and substantia nigra (SN) (Fig 1A), periglomerular neurons in the olfactory bulb (OB) (Fig 1B) [21] and noradrenergic neurons in the locus coeruleus (Fig 1C) [22], suggesting that no alteration of *Th* expression pattern resulted from the insertion of IRES-Cre cassette. To further confirm this at single-cell level, we examined the relative expression pattern of *Th* to *DAT*. In wild type rats, DAT co-expresses with Th in dopaminergic neurons of VTA and SN (Fig 1D–1D”). Due to the lack of high quality anti-rat DAT antibodies, we performed fluorescent RNA *in situ* hybridization (RNA-FISH) for DAT, followed by anti-TH immunostaining (flowchart shown in S1A Fig). Exactly as in the wild type rat brain, the TH protein in the brain of homozygous Th-Cre rats co-localized precisely with the mRNA of DAT in VTA and SN, including the compacta dorsal tier (SNCD), the reticular part (SNR) and the lateral part (SNL) (Fig 1E–1E”; 1F–1F”). These data demonstrate that the insertion of the IRES-Cre cassette does not alter the expression pattern of the *Th* gene.

### The expression pattern of the Cre recombinase recapitulates that of the endogenous Th gene

**Fig 2:**
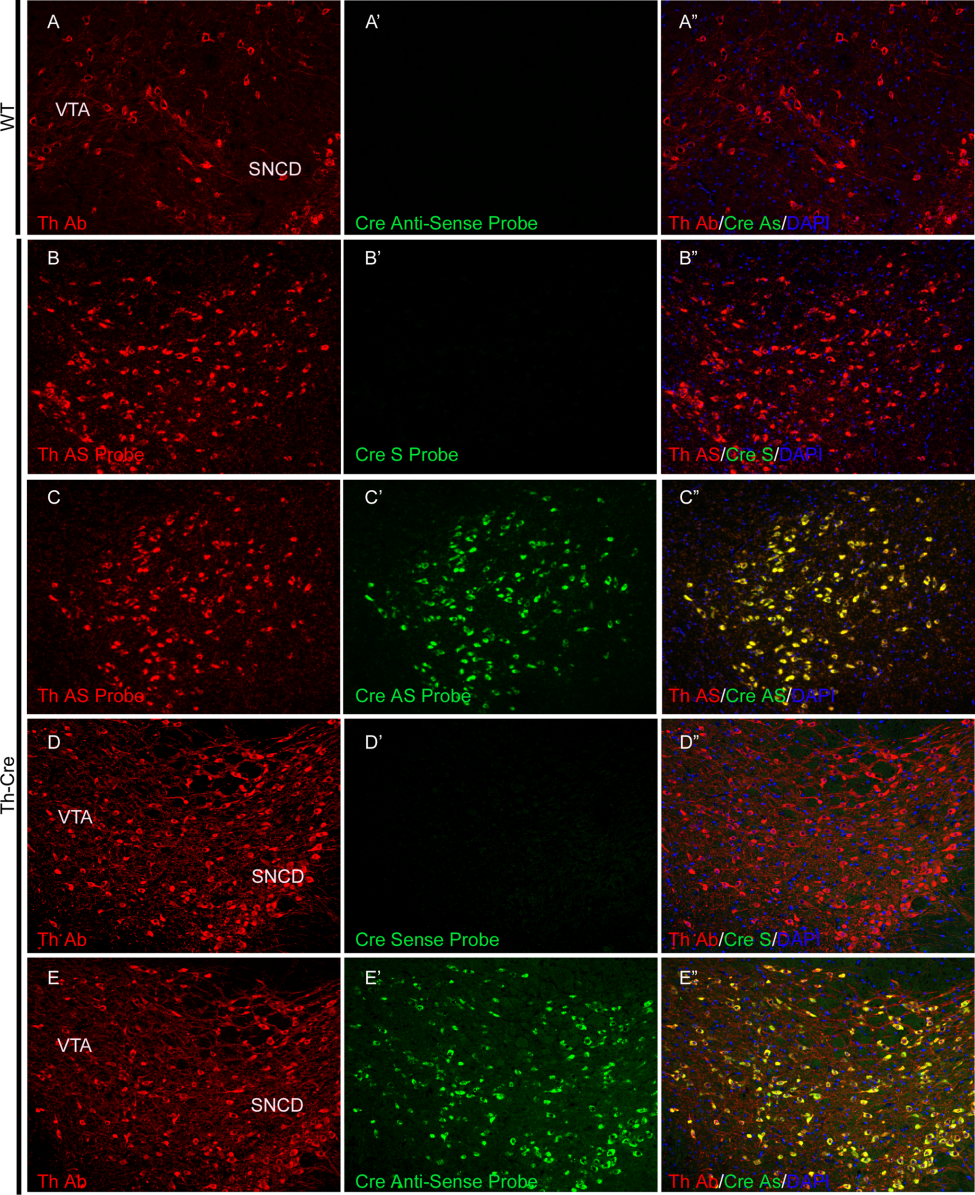
The expression of Cre recombinase recapitulates that of Th in dopaminergic neurons in the ventral tegmental area and substantia nigra. A combination of anti-TH immunostaining and Cre fluorescence *in situ* hybridization (A-A”, D-E”) as well as double fluorescence *in situ* hybridization with both Th and Cre riboprobes (B-C”) were employed to examine the co-localization of Cre mRNA and the Th gene in dopaminergic neurons of ventral tegmental area and substantia nigra. SNCD, substantia nigra compacta dorsal tier; VTA, ventral tegmental area; S probe, sense probe; AS probe, anti-sense probe.

To test whether Cre expression only occurs in Th-positive cells in the adult brain, we performed immunostaining of TH and RNA FISH for Cre, for the lack of a good anti-Cre antibody. As expected, no Cre mRNA expression is observed in the brain of wild type rats (Fig 2A–2A”; S2A–S2A” Fig). In contrast, on Th-Cre brain sections, we observed complete co-localization of Th mRNA and Cre mRNA in a double RNA FISH (see S1B Fig for basic experimental procedure) in VTA and SN (Fig 2C–2C”) as well as by coupling immunostaining with an anti-TH antibody and RNA FISH on Cre (Fig 2E–2E”). Co-localization of Th and Cre mRNA was observed in other Th-expressing areas in the brain of Th-Cre rats, including the olfactory bulb and LC (S2 Fig).

**Fig 3:**
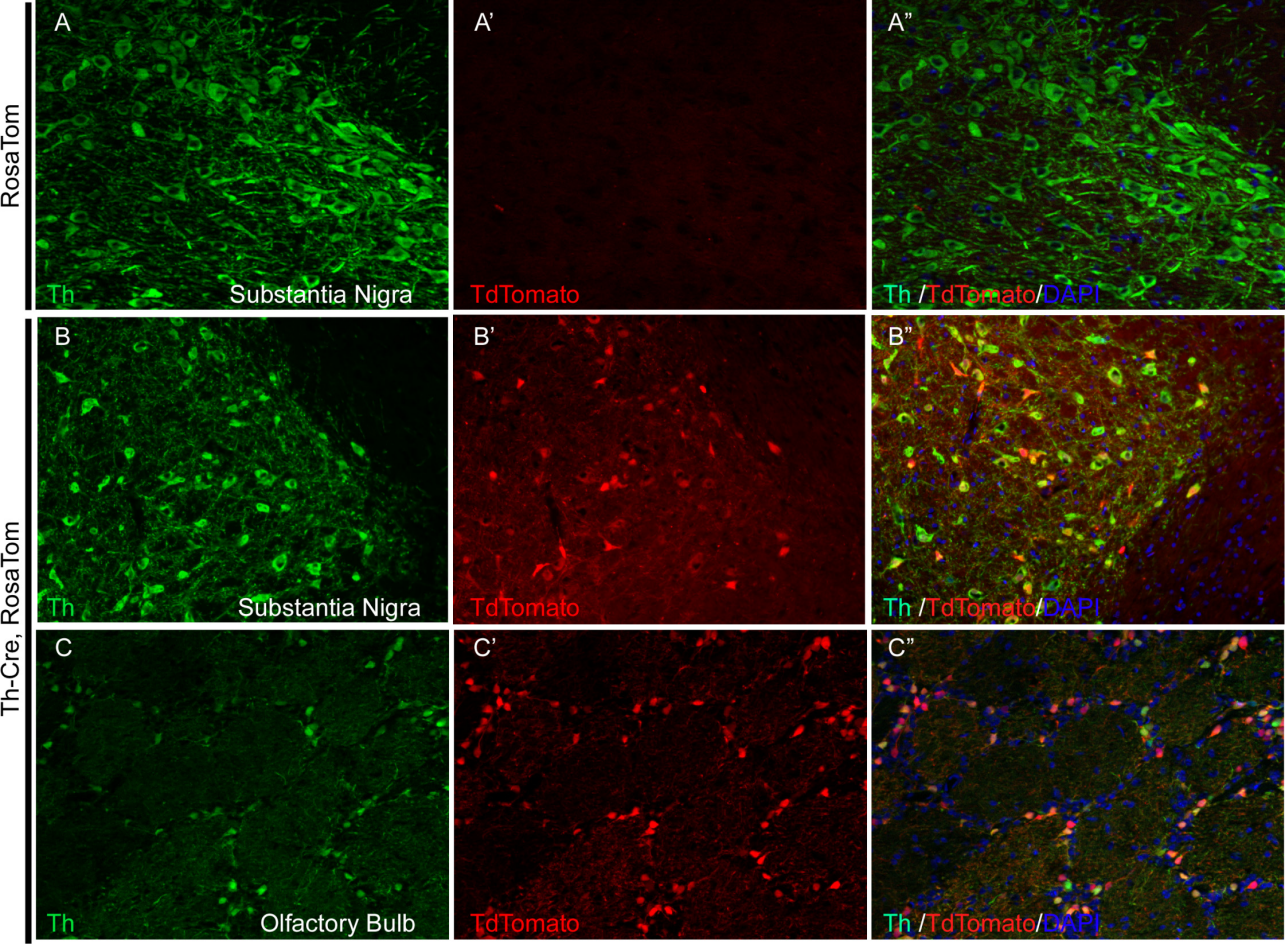
Tdtomato labels Th positive cells in the substantia nigra area of Th-cre, Rosa Tom rat brain. (A-A”), anti-TH antibody staining was done on Rosa Tom brain sections and no live tdTomato signal could be detected; In contrast, in both SN (B-B”) and OB (C-C”), co-localization of TH antibody signal and live tdTomato signals could be observed.

To simplify the detection of Cre expression, we decided to establish a fluorescent reporter rat. We first created SageR by inserting into the Rosa26 locus a human PGK promoter-driven turboGFP open reading frame preceded by a floxed stop cassette, where GFP expression was expected to be activated upon Cre-mediated excision of the floxed stop cassette. Even though we detected excision on the DNA level, we were unable to detect GFP expression upon Cre mediated excision although excision was detected on the DNA level (not shown). We then generated another reporter line, the Rosa Tdtomato (Rosa Tom), in which the expression of Tdtomato is under the control of a ubiquitous CAG promoter after Cre-mediated excision of the preceding floxed stop cassette, similar to the Ai14 reporter mice[23] (detailed description of this line will be reported in another manuscript). With Rosa Tom line, we observed co-localization of Th antibody signal with Tdtomato expression in the dopaminergic neurons in both olfactory bulb, VTA and SN (Fig 3). In addition, we noticed that some Th negative neurons are also labeled with tdtomato in the olfactory bulb, cerebral cortex and hypothalamus (S3 Fig). Finally, we investigated the labeling pattern of Th-cre outside of brain and found a few highly scattered Tdtomato postive cells in various adult organs, including kidney, heart, liver and thymus, but not spleen (S4 Fig).

**Fig 4:**
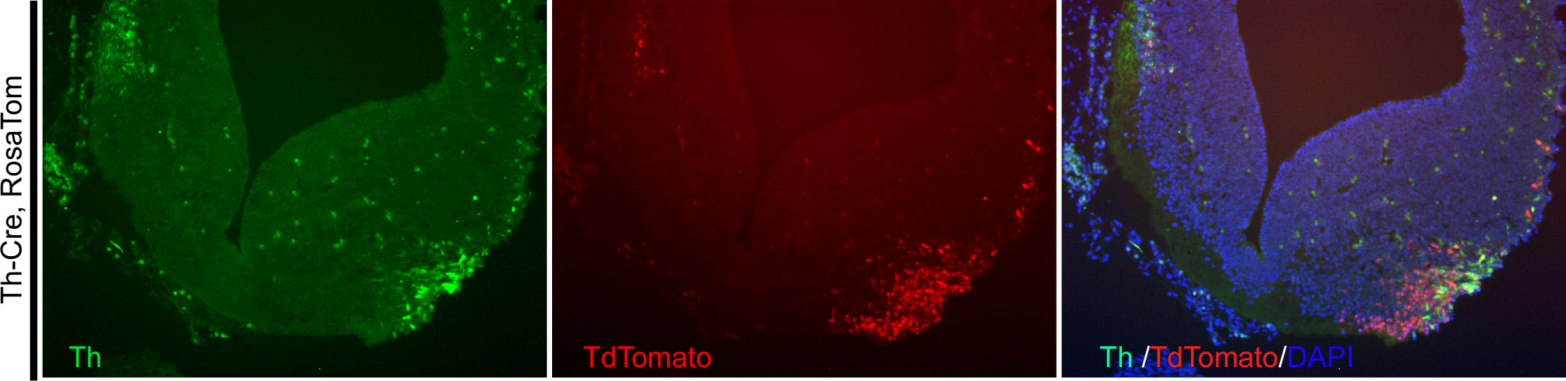
Cre labeling pattern in the developing midbrain of 13.5 day old Th-cre, Rosa Tom embryos is consistent with expected Th expression. E13.5 Th-cre, Rosa Tom embryos were analyzed for co-localization of Th and Tdtomato in the substantia nigra neurons of the developing mid-brain. Note that newly emerged, not fully differentiated neurons that are in the process of migrating away from ventral peri-ventricular surface have weak expression of Th and are barely labeled with Tdtomato since the excision of the floxed stop cassette, the translation, expression and maturation of Tdtomato takes time. In contrast, well-differentiated substantia nigra neurons located at the surface of the lateral side of the midbrain express higher levels of Th and are strongly labeled with Tdtomato.

To investigate the temporal labeling pattern of Th-cre, we compared the expression of Th and Tdtomato in the developing midbrain of 13.5 day old Th-cre, Rosa Tom embryos (Fig 4). In the developing midbrain, neuroblast cells located near the ventricular surface divide and give birth to immature neurons, which start differentiation when migrating away to the lateral side. Therefore, the undifferentiated dopaminergic neurons express very low amount of Th before it reaches the lateral side of the ventral midbrain and are barely labeled with tdTomato expression. This delay of tdTomato labeling is an inherit properties of the Cre activity reporter since the excision of the floxed stop cassette, the transcription and translation of tdTomato could only start after Cre (or Th) expression[24]. In contrast, well-differentiated cells that are located near the lateral surface express higher level of Th and are strongly labeled with tdTomato (Fig 4).

### The creation and characterization of another dopaminergic neuron-specific Cre line, DAT-Cre

**Fig 5:**
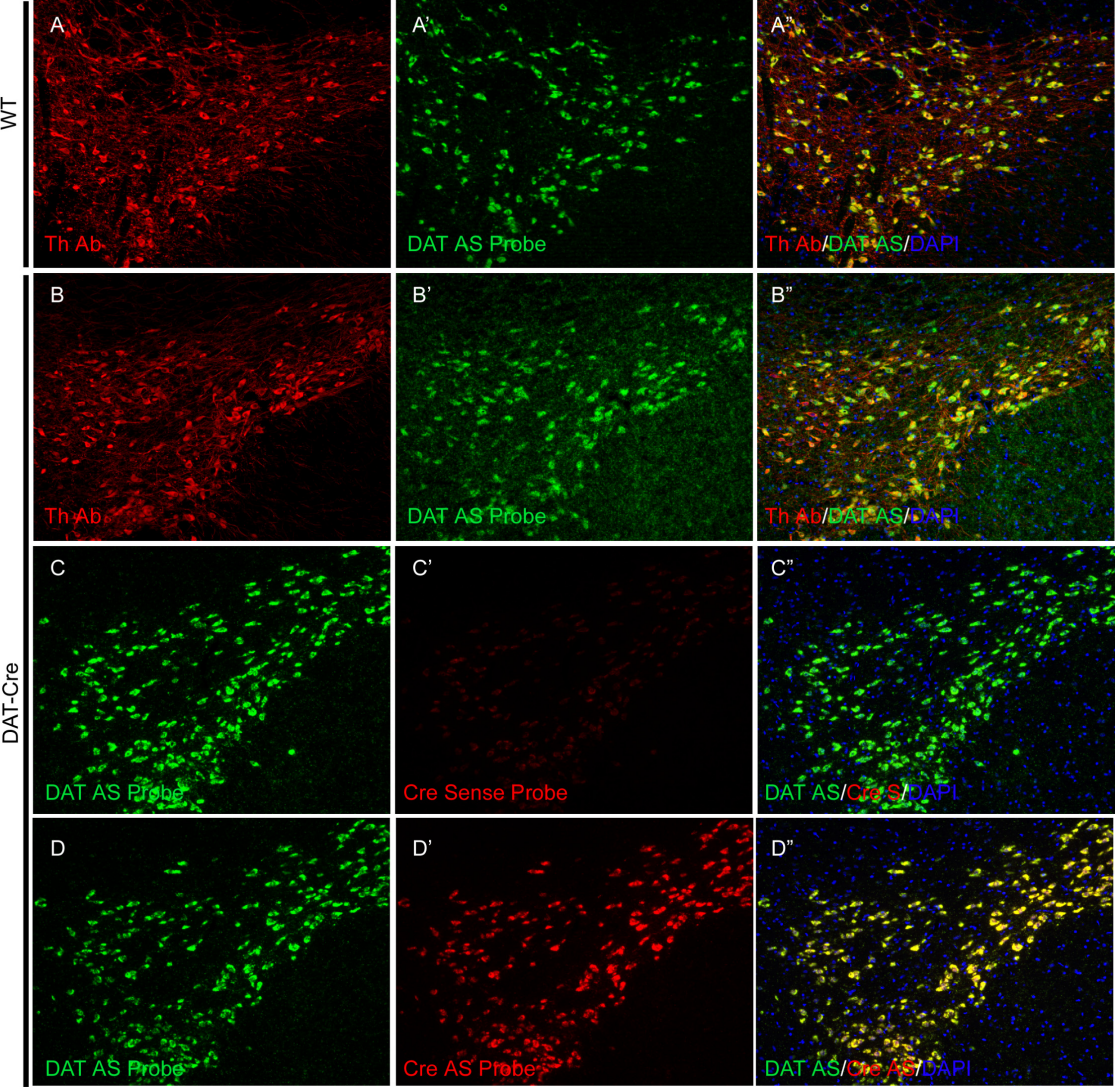
The Cre recombinase in DAT-Cre rats faithfully recapitulates the expression pattern of endogenous DAT. (A-A”), anti-TH antibody and DAT RNA *in situ* hybridization on wild type rat brain sections; (B-B”), similar staining on DAT-cre homozygous rat brain sections. (C-D”), double *in situ* hybridization of Th and DAT on DAT-cre homozygous rat brain sections. AS, anti-sense probe.

**Fig 6:**
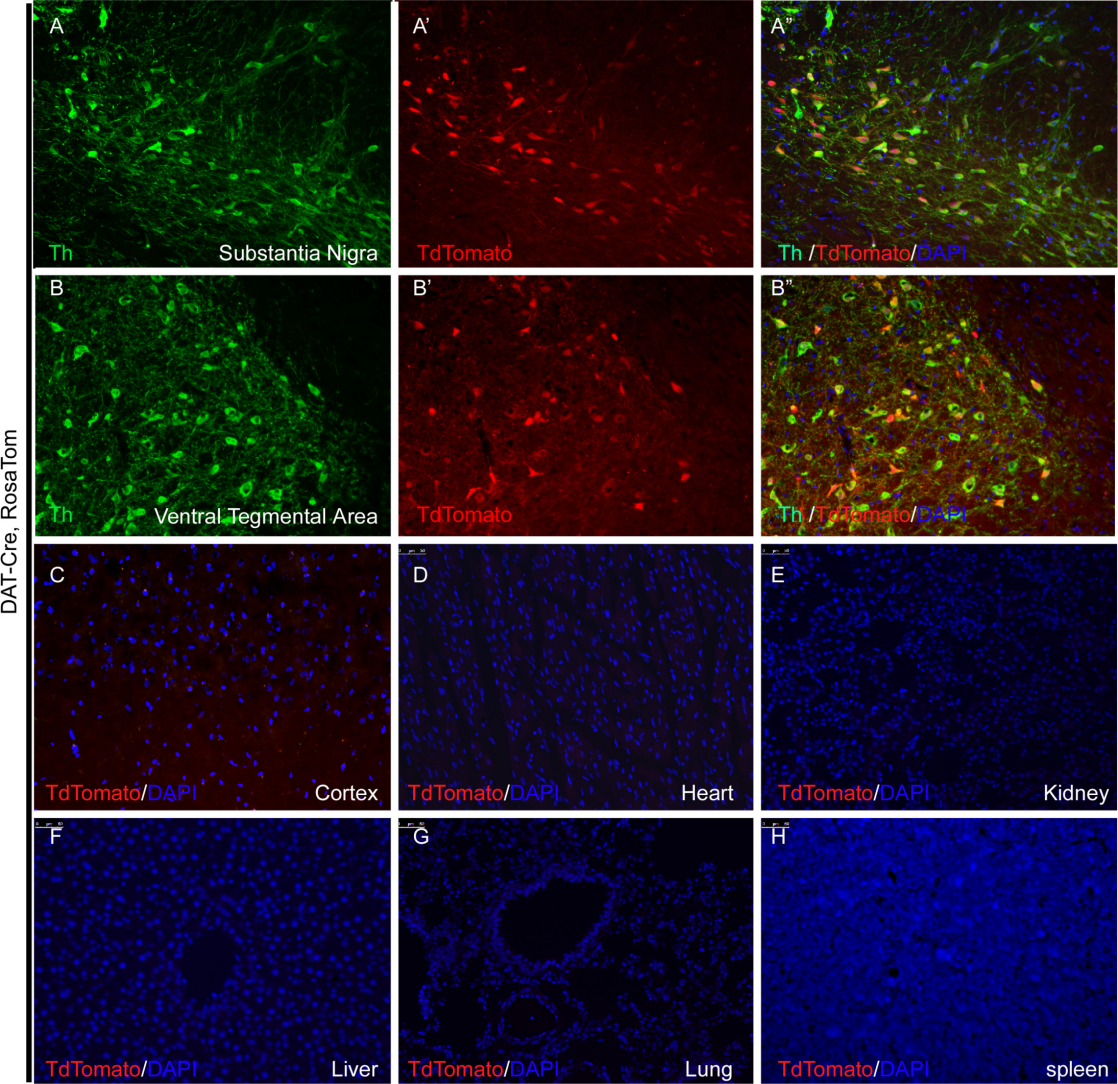
In DAT-cre, Rosa Tom rats, only Th-positive dopaminergic neurons in the SN and VTA, but not any other cells in the brain or other adult organs, were labeled with tdTomato. (A-B”) shows overlapping signals between TH antibody staining and live tdTomato signal in the dopaminergic neurons of the SN (A-A”) and VTA (B-B”) area. In contrast, no live tdTomato signal could be detected in other brains areas such as cerebral cortex (C) and other adult organs including heart (D), kidney(E), liver (F), lung (G) and spleen (H).

To complement the Th-cre line, we created a DAT-Cre line using a similar knock-in strategy (S5 Fig) The expression of endogenous dopamine active transporter (DAT) largely co-localizes with Th in dopaminergic neurons of ventral tegmental area and substantia nigra in the midbrain [25, 26] (Fig 3A). Indeed, in both wild type and homozygous DAT-cre rats, TH immunostaining and DAT *in situ* hybridization shows nice co-localization of the two dopaminergic neuron marker genes, indicating that the insertion of the IRES-Cre cassette did not change the expression pattern of endogenous DAT gene (Fig 5, compare B-B” to A-A”). Furthermore, double *in situ* hybridization of DAT and Cre revealed complete co-localization in dopaminergic neurons in ventral tegmental area and substantia nigra (Fig 5D–5D”). And more importantly, when this DAT-Cre line is mated with Rosa Tom, tdTomato positive cells could only be observed in the Th positive neurons in the VTA and SN, but not any other cells in the brain or other adult tissues examined, including heart, kidney, liver, lung, spleen and thymus (Fig 6), confirming that the expression of the Cre recombinase faithfully recapitulates the expression pattern of endogenous DAT.

### Th-Cre is capable of excising floxed allele in dopaminergic neurons in the brain *in vivo*

**Fig 7:**
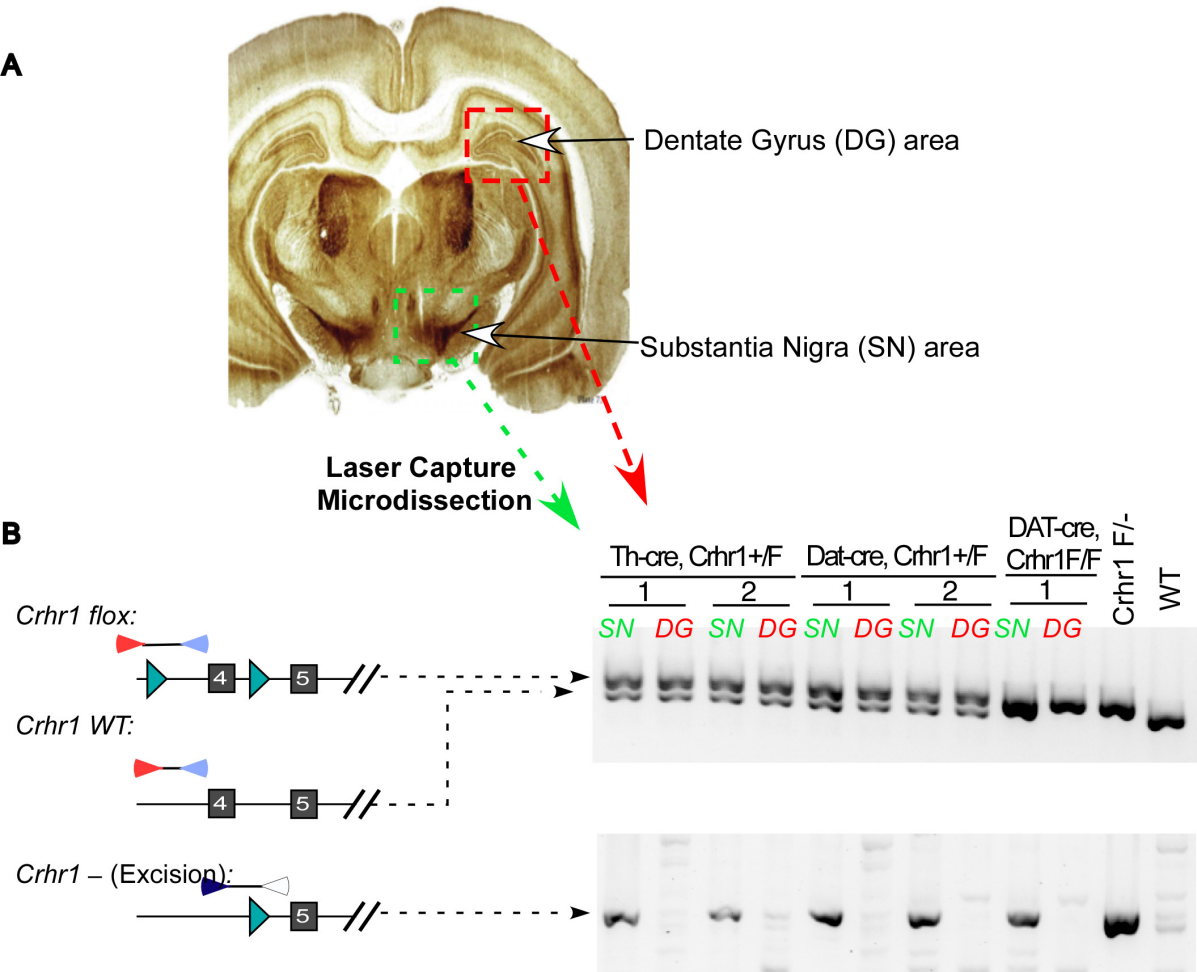
Both Th-Cre and DAT-Cre were functional and excised floxed alleles in expected cells *in vivo*. Tissue region that includes Cre-expressing neurons (SN, substantia nigra area) is demarcated with green square, and red square, non-dopaminergic neurons (HC, himpcampus area) (A). Samples from these regions were collected using laser-assisted microdisection and lysed for PCR analysis with primers that specifically detect the wild type (WT), floxed and excised Crhr1 locus (B). Similar analyses were done on rats with three different genotype: Th-cre/+, Crhr1 +/f; DAT-cre, Crhr1 +/f and DAT-cre, Crhr1 f/f. Note that the floxed Crhr1 PCR band in SN sample from DAT-cre, Crhr1 F/F runs a little faster due to the presence of a large amount of PCR product.

To demonstrate that the floxed alleles are efficiently excised in Th-positive neurons in Th-Cre rats, we mated Th-Cre male rats with female rats that harbor floxed *Crhr1* gene (Corticotropin-releasing hormone receptor 1) and obtained pups with the genotype of Th-Cre/+, Crhr1 +/flox. Likely because of the extremely small fraction of neurons being Th-positive, we were not able to detect excision in whole brain homogenate. So we prepared freshly frozen brain sections and collected VTA/SN and hippocampus areas (dentate gyrus) using laser capture microdissection (LCM), respectively (Fig 7A). Excision of the floxed allele was readily detected by using PCR amplification of the genomic DNA prepared from the Th-positive VTA/SN samples but not samples from dentate gyrus, which is known to be Th-negative (Figs 1A and 7B). It is worth noting that due to the contamination of Th-negative cells in the samples, we could not assess the completeness of excision of the floxed allele. We attempted to answer this question by other means such as immunostaining for the disappearing of Crhr1 protein and FISH for the loss of Crhr1 mRNA in Th-cre, Crhr1 Flox/Flox rats without success due to either the lack of good antibodies or the low abundance of Crhr1 mRNA transcripts (data not shown).

Similarly, PCR analyses following laser-assisted microdissection of DAT-cre, Crhr f/+ or DAT-cre, Crhr1 f/f rat brains prove that DAT-Cre excises the floxed allele in specific neurons (Fig 7B). In summary, these results show that both Cre lines are sufficient to excise floxed alleles in specific sets of dopaminergic neurons.

### Th-cre mediates complete germ line excision of the floxed allele when maternal rat harbors both Th-Cre and floxed alleles

The expression of *Th* gene is known and also confirmed by our immunostaining to be largely limited to dopaminergic and noradrenergic neurons in the central nervous system, sympathetic neurons of the peripheral nervous system, and the medulla of adrenal gland [22, 27]. However, in the process of crossing the Th-Cre line and floxed Crhr1 line, we observed that when female rats harboring both Th-cre and floxed alleles (for example, Th-Cre/+, Crhr1 +/flox) were mated with wild type males, no pups with floxed allele were obtained; instead, all pups carried either a wild type or excised allele (Table 1). This is in stark contrast to offspring from male rats that harbors Th-cre and a floxed allele (Table 1) or female rats that harbors DAT-cre and floxed alleles (data not shown), where no excision of floxed allele could be observed during genotyping using either clipped tail or ear samples. No germ line excision was detected in offspring between female rats harboring only Th-Cre allele and male rats that harbored floxed allele, indicating that Th-cre is not active in fertilized eggs (Table 2). We further found that when Th-Cre, Crhr1+/floxed female rats were mated with wild type males, not all pups with excised allele contain a Cre allele (Table 3), suggesting that Cre is expressed before the end of meiosis I during oocyte formation, i.e., before the segregation occurs between chromosome 7 (harboring Th-cre allele) and 17 (harboring Crhr1 allele) (S6 Fig Scenario A), but is absent in fertilized eggs. In contrast, we did not observe any germ line Cre activity with either male or female DAT-cre rats (Table 1).

**Table 1:** Germ line excision of floxed alleles in maternal, but not paternal, Th-Cre rat.

Mother	Father	No. of total pups (No. of matings)	No. of pups with floxed allele	No. of pups with excised allele
Th-cre/+, Crhr1 +/Flox	WT	51 (3)	0	18
WT	Th-cre/+, Crhr1 +/flox	27 (2)	19	0
WT	Th-cre/+, SageR+/flox	8 (1)	7	0
WT	DAT-cre/+, Crhr1 +/Flox	39 (1)	20	0
WT	DAT-cre/+, SageR+/flox	50 (2)	23	0

**Table 2:** Th-cre mediated excision of floxed allele does NOT occur in fertilized oocytes.

Mother	Father	No. of total pups (No. of matings)	No. of pups with floxed allele	No. of pups with excised allele
Th-cre/+	Crhr1 +/Flox	14 (1)	8	0
Th-cre/+	SageR +/flox	11 (1)	8	0

**Table 3:** The excision of floxed alleles in the oocyte occurs before Meiosis I during occyte development.

No. of total pups	No. of Th-cre/+, Crhr1 +/floxed pups	No. of Th-cre/+, Crhr1 +/- pups	No. of Crhr1 +/- pups	No. of Crhr1 +/flox pups	No. of pups with other genotype
Litter 1: 11	0	5	2	0	4
Litter 2: 13	0	3	1	0	9
Litter 3: 14	0	1	3	0	10
Litter 4: 13	0	1	2	0	10

## Discussion

Tissue/cell type-specific gene disruption is essential to the dissection of gene functions. The Cre-loxP system has been extensively used in mouse genetics, accumulating hundreds of Cre lines (for repository of mouse Cre lines, please see http://cre.jax.org/strainlist.html; http://nagy.mshri.on.ca/cre_new/index.php). In contrast, only a very limited number of rat Cre lines have been created [28–32] and most of these are based on random insertion of a Cre transgene. Although relatively simple, such transgenic approach often suffers from a number of drawbacks: First, the promoter used for driving the expression of the Cre gene is often truncated, even when on a BAC (bacterial artificial chromosome) or PAC (P1-derived artificial chromosome), and therefore may not include all essential regulatory elements to recapitulate the exact expression pattern of the endogenous promoter. In fact, in a Th-Cre rat created by using random transgene, only about 85% of Th-positive neurons expressed Cre and vice versa [29]. BAC transgenes also often introduce extra copies of adjacent genes to the genome, which may complicate phenotypic analysis. Second, the random nature of insertions renders the expression of Cre transgene susceptible to the positional and epigenetic silencing effects. In contrast, site-specific insertion of a Cre cassette to be under a given promoter in its endogenous locus has been shown in the mouse to lead to complete identical expression profile between Cre and the target gene [33]. The development of programmable nucleases allows efficient targeted integration in practically any species, without the use of embryonic stem cells [14, 34]. We reported previously the creation of TH-Cre rats using ZFN technology (Brown et al. 2011). Here we present the generation of DAT-Cre rats and the characterization of Cre expression patterns in the brains of both TH-Cre and DAT-Cre rats. We expected that in both Cre lines, the expression of the Cre recombinase could recapitulate that of the endogenous gene to which they are placed after, and were limited to the dopaminergic neurons in the midbrain. Indeed, we observed that the expression of Cre in both lines mostly reflects the expression pattern of either Th or DAT in the adult brain, although we did observe Cre expression sporadically in Th negative neurons in the olfactory bulb, cerebral cortex and hypothalamus as well as some cells in the other adult organs. We reason that these might be progeny of TH-positive cells earlier in the development that no longer express TH. Temporary presence of Cre in given cells during earlier development leads to permanent excision of floxed stop in the reporter cassette at the DNA level, which is maintained in all progenies from those cells. In other words, the status of the reporter in a given cell is the result of accumulation of previous Cre activity throughout the cell’s lineage whereas Th antibody staining could only detect its expression at the time of experiment[24, 35]. On the other hand, without an extensive analysis of TH expression at every stage, we also cannot exclude the possibility that targeted insertion of the IRES-Cre contributed to ectopic expression earlier in development.

Interestingly, the TH-Cre rats also exhibited Cre activity in the female germ cells, as evidenced by the complete loss of floxed allele in the offspring when the maternal genotype has both Th-cre and floxed alleles. Furthermore, the fact that when Th-cre/+ female was mated with Crhr1+/Flox or SageR +/flox male, no complete loss of the floxed allele was detected in any offspring indicates that Cre is not present/active in the fertilized eggs. Instead, the presence of offspring with complete loss of the floxed allele but no Cre (Crhr1 +/-) from matings between female Th-cre/+, Crhr1+/Flox and wild type male strongly argues that the expression of Cre must have occurred before the segregation between the Cre allele and the floxed allele during meiosis of the oocyte, i.e., before meiosis I. Otherwise, if the expression of Cre protein occurs after meiosis I, then only oocytes that have both Cre and floxed Crhr1 allele could have excised Crhr1 allele and those that only have floxed allele but not Cre will not undergo any excision of the Crhr1 allele (S6 Fig Scenario B), which is inconsistent with our observation. As the excision of the floxed alleles reflects the history of the Cre activity, it is challenging to determine when exactly the Cre recombinase is expressed during oocyte/embryo (of the mother) development. It is possible that the germ line expression of Cre recombinase reflects the expression of endogenous Th gene. However, without following TH expression pattern in the process of gamete formation in wild type female rats, we cannot exclude the possibility that the Cre activity in oocyte development is ectopic, which has been observed with some other Cre lines in mice [36, 37](and personal observations). Nevertheless, we do not expect that the presence of such Cre activity in female germ cells will affect the application of the Th-cre rats. On one hand, careful design of mating scheme could avoid such germ line excision (for example, by mating female Crhr1 Flox/Flox with male Th-Cre/+, Crhr1 Flox/Flox); on the other hand, this phenomenon makes Th-Cre a useful germ line deleter to derive whole body knockout rats from the conditional ones.

We envision that in addition to drive conditional knockout of specific genes, these lines will be highly useful in the emerging optogenetics field, especially along with Cre-dependent opsin-expressing rats, in which the expression of excitatory or inhibitory rhodopsin will only be expressed upon Cre-mediated excision [23]. We believe that the combination of these lines could offer powerful optogenetics tools for effective study in rat models. More excitingly, thanks to the rapid development of programmable nuclease technologies, in particular the advent of the CRISPR/Cas9 technology, similar conditional and Cre knock-in rats can now be created in a relatively short time [31, 38] (Liu and Cui, manuscript in preparation). A large repertoire of Cre rats will be invaluable and much needed for neuroscience research.

A huge challenge when working with non-popular models like rats is the lack of high quality antibodies. Here we show that this could be circumvented most time by utilizing fluorescence mRNA *in situ* hybridization, which could be combined with immunohistochemistry, if good antibody is available for one of the antigen under the study, or another anti-sense riboprobe labeled with different hapten or biotin (double FISH). In particular, the adoption of the TSA PLUS system (S1 Fig) could dramatically enhance the sensitivity and make it a plausible method to detect the expression of low abundance of mRNA. One should note that the high hybridization temperature for riboprobes (65–70°C) may kill live fluorescent protein signal, therefore anti-EGFP or anti-tdTomato antibody staining is required to visualize their signal when combined with mRNA *in situ* hybridization.

## Materials and Methods

### Ethics statement

All rat work was performed in accordance with the approved animal protocols overseen by SAGE’s Institutional Animal Care and Use Committee (IACUC). Sprague Dawley rats (Ntac:SD) from Taconic Farms (Hudson, New York) were used for both microinjection and breeding and were housed in standard cages and maintained on a 12 h light/dark cycle with *ad libitum* access to food and water. Routine health monitoring of the colony was performed at IDEXX (Columbia, MO) and revealed no evidence of infection with known serious pathogens.

### Microinjection

Four to five week-old donors were injected with 20 units of PMS followed by 50 units of hCG injection after 48 h of the PMS injection and then immediately mated with stud males after the hCG injection. Fertilized eggs were harvested a day later for microinjection. Following microinjection, 25–30 eggs were transferred into each pseudopregnant female, which gave birth to the founder generation.

### ZFN and donor construction

The creation of Th-Cre and floxed Crhr1 rats were described in [20]. To create DAT-Cre rats, a pair of ZFNs targeting close to the translational stop codon was first validated with the following target sequence: 5’agctgcgtcactggctgttgctgtaaagtggaaggagacagct3’. To construct the donor, two 800 bp of sequences immediately flanking the ZFN cleavage site were PCR-amplified from genomic DNA and cloned into pBS vector into KpnI and SacII sites. The IRES-Cre cassette was then inserted into NotI and AseI sites between homology arms. The whole construct was sequence confirmed. Sprague Dawley rats were co-injected with ZFN mRNA (10 ng/ul) and donor (1 ng/ul, supercoiled plasmid).

We have tried to create two reporter for analyzing Cre activity. In one reporter line, SageR, we site-specifically inserted a floxed stop TurboGFP cassette to the Rosa26 locus in the rat. A pair of ZFNs were validated to target the following sequence with high efficiency: 5’agactccagttgcagatcacgagggaagaagggggaagg3’. To construct the donor, we first cloned two 800 bp homology arms flanking the target site into the pBluescript vector between the KpnI and SacII, and then inserted a cassette containing the human PGK promoter, loxP-3x SV40 poly A-loxP, turbo GFP (Evrogen) and BGH poly A signal, into BamHI and NheI sites between the homology arms. The resulting donor plasmid, named as SAGE-R (stands for SAGE reporter) was treated with recombinant Cre protein *in vitro* to excise 3x pA between the loxP sites, resulting Rosa GFP donor, which was transfected into rat C6 cells to confirm the Turbo GFP expression. SAGE-R reporter line was created by coinjecting Rosa26 ZFN mRNA (10 ng/ul) and SAGE-R donor (1 ng/ul, supercoiled plasmid) into single cell embryos of Sprague Dawley rats. Unfortunately, although robust expression of TurboGFP could be observed in cell culture, no live TurboGFP signal could be detected in SageR rat after Cre-mediated stop cassette excision due to unknown reasons (data now shown). Another reporter line, Rosa tdTomato (Rosa Tom), was created witih CRISPR/Cas9 technology and worked as expected. The details of its creation and characterization will be described in details in a different manuscript.

### Immunohistostaining

Immunohistostaining was performed as described in [39] with modifications. Briefly, adult rats were anesthetized with KAX (86.9 mg/ml Ketamine, 0.43 mg/ml Acepromazine and 5.22 mg/ml Xylazine) at a dosage of 1 μl/gram body weight through intraperitoneal injection and then perfused with 10% neutral buffered formalin (NBF) (Sigma-Aldrich). Brains were dissected and post-fixed in 10% NBF overnight, thoroughly washed and soaked in 30% sucrose in 1XPBS until they sank. They were then embedded into Optimal Cutting Temperature compound (Tissue-Tek) and cut into 8μm thick frozen sections. Before incubated with primary antibodies, brain sections were permeabilized with 0.1% Triton X-100 in 1XPBS at room temperature for 20 minutes and blocked in Blocking buffer (3% BSA, 0.1% tween in 1XPBS) for 1 hour. Antibody incubation was performed at 4°C overnight. Mouse anti-tyrosine hydroxylase (clone LNC1, Millipore) monoclonal antibody was used at 1:400. Following washing steps with 1XPBS, Alexa Fluor 488 or Cy3-conjugated anti-mouse secondary antibody (Jackson ImmunoResearch) was used to detect the signal of the primary antibody. For Rosa Tom reporter, the live tdTomato signal was acquired with Cy3 filter. At least three animals from each genotype were analyzed.

### *In situ* hybridization

*In situ* hybridization was essentially performed as described in [40], however, fluorescence-, instead of chromogenic-, based signal detection method was used to ease the co-localization study. Briefly, frozen brain sections were prepared in a similar way as those in the immunohistostaining method, except that caution was taken to avoid RNase-contamination. After digestion with proteinase K to facilitate probe penetration, inactivation of endogeneous peroxidase activity with 3% H_2_O_2_ and treatment with acetylatioin solution to reduce non-specific hybridization, sections were then blocked with hybridization solution (10mM Tris, pH7.5; 0.6M NaCl; 1mM EDTA, pH 8.0; 0.25% SDS; 1XDenhardt’s; 0.2mg/ml yeast tRNA; 50% formamide), followed by hybridization with denatured probes at 65°C overnight. For single probe *in situ* hybridization, including the ones that are combined with immunostaining, probes were labeled with Digoxigenin-11-UTP (Roche Applied Science) with MaxiScript *in vitro* Transcription Kit (Ambion). After stringent wash, probes were detected with horseradish peroxidase-conjugated anti-Digoxigenin antibody followed by TSA Plus Cyanine 3 or Fluorescein System (PerkinElmer). For experiments with a combination of *in situ* hybridization and immunostaining, mouse anti-Th antibody was included when the sections were incubated with horseradish peroxidase-conjugated anti-Digoxigenin antibody after probe hybridization and Cyanine 3 conjugated-anti-mouse secondary antibody was used for the signal detection (S1A Fig).

For double probe *in situ* hybridization, one probe is labeled with Digoxigenin-11-UTP and the other with Fluorescein-12-UTP (Roche Applied Science) (S1B Fig). Whereas both probes were hybridized in the same step to their targets in the sections, the signals were detected sequentially. Since the signal from Fluorescein labeled probe is generally weaker, this was detected first with two rounds of amplification: after stringent wash to remove extra non-hybridized probes, sections were incubated with peroxidase-conjugated anti-fluorescein antibody (Roche Applied Science), which, upon incubation with TSA Plus Fluorescein substrate in the next step, catalyzes the deposition of fluorescein; and in the second round of signal amplification steps that follow, the newly formed fluorescein was again detected with peroxidase-conjugated anti-fluorescein antibody, which subsequent catalyzes the deposition of more fluorescein when TSA Plus Fluorescein substrate is present. Before the signal from the Digoxigenin-labeled probe was detected, sections were treated with 3% H_2_O_2_ in 1XPBS for 30 minutes. Similar to the detection of the signal from Fluorescein-labeled probe, the signal from Digoxigenin-labeled probe was then detected by incubating sections with peroxidase-conjugated anti-Digoxigenin antibody and TSA Plus Cyanine 3 substrate (PerkinElmer). Importantly, control sections either without peroxidase-conjugated anti-Digoxigenin antibody or without Digoxigenin-labeled probe were included to ensure the complete elimination of peroxidase activity derived from anti-fluorescein antibody that was used to detect the Fluorescein-labeled probe. At least three animals from each genotype were analyzed.

### Image capture and processing

Images were captured with ApoTome Microscope (Zeiss) and processed with Image J, Adobe Photoshop CS2 and Canvas X.

### Laser-assisted microdissection

Laser-assisted microdissection was performed as described in [41]. Briefly, brains were dissected from euthanized rats and freshly frozen on dry ice. 30μm sections were cut with Leica Cryostat, treated with serial ethanol (100%, 95%, 75%, 50%), stained with Toluidine blue and dehydrated with serial ethanol (50%, 75%, 95%, 100%) and xylene substitute. Microdissection was performed with Leica LMD6000 Laser Microdissection System. Microdissected samples were lysed with QuickExtract DNA Extraction Solution (Epicentre) for PCR analysis.

### Genotyping and PCR excision analysis

Th-Cre, conditional Crhr1, Grin1 rats were genotyped as described in [20]. The following primers are used for genotyping: DAT 3’F3 (5’ATACCGGAGATCATGCAAGC3’) and DAT 3’R2 (5’GAGCAGGTGTCCAGAAAGGTG3’) for DAT-Cre, expected size around 1kb; Rosa900 5’F (5’GAGAAGGGAGCGGAAAAGTC3’) and RosaGFP 5’R (5’CGGACGTGAAGAATGTGCGAG3’) for SageR genotyping, expected size 1030bp; To assess the excision status of the SageR locus, the following primers were used for PCR: SageR EX-1F (5’ GTGTTCCGCATTCTGCAAGC3’) and SageR EX-1R (5’ gtgatgcggcactcgatctc3’). Such primer pair yields a 245bp band for excised allele and a 665bp band for non-excised allele. To assess the excision status of the Crhr1 allele, two different PCR reactions were performed: one with primers Crhr1 int3 out-F (5’ AGACCCCTAGAGAGGTTTCTGTCTGC3’) and Crhr1 int3 out-R(5’TTCTCTTTGGAACTACTGGGTGAGC3’) that will amplify the un-excised floxed or wild type allele; another with primers Crhr1 ex-2F (5’ tgaaggtgtggtaggtcatc3’) and Crhr1 ex-2R (5’ catctgtggagctgatgctg3’) that will amplify the excised allele.

## Supporting Information

S1 Fig Flow charts for combined of FISH and IHC (A) or double probe FISH (B). Note that two rounds of tyramide signal amplification (TSA) were employed to enhance sensitivity in B. Ab, antibody; Cy3, Cyanine 3; DIG, Digoxigenin; FITC, fluorescein; POD, peroxidase.
(TIF)

S2 Fig The expression pattern of Cre recombinase in the olfactory bulb and locus coeruleus. Double staining with Th antibody (red) and cre anti-sense probe (green) revealed their co-localization in the olfactory bulb (C-C”) and locus coeruleus (D-D”). OB, olfactory bulb; LC, locus coeruleus; S probe, sense probe; AS probe, anti-sense probe.
(TIF)

S3 Fig TdTomato labels some Th negative cells in the brain of Th-cre, Rosa Tom rats. (A) In addition to Th positive neurons, some Th negtive cells in the olfactory bulb are also labeled (Anterior is on the left). A few other Th negative cells were also observed in some other brain areas, including cerebral cortex (B) and hypothalamus (C), but not in the dentate gyrus (D).
(TIF)

S4 Fig TdTomato labels a few Th negative cells in some other organs in adult Th-cre, Rosa Tom rats. No tdTomato labeled cells were found in any adult organs of RosaTom rat (A-E), confirming that there is no leakage expression of tdTomato reporter in the absence of Cre activity. In contrast, a few tdTomato labeled cells could be observed in some adult organs of Th-Cre, RosaTom rats, including kidney (G and H), heart (I), liver (J and K) and thymus (L). No labeled cells could be observed in the spleen (F). It is unclear whether the labeling reflects ectopic Cre expression or historic transient expression of Th gene.
(TIF)

S5 Fig Schematic of IRES-Cre insertion into the *DAT* locus. Top panel shows the wild type DAT locus and the bottom panel, with IRES-Cre inserted immediately after the translational stop codon of *DAT* gene.
(TIF)

S6 Fig Th-cre must be expressed in female germ cells before the end of meiosis 1. Th-cre is located on chromosome 7 whereas Crhr1 gene is on chromosome 17. And for simplicity, only these two pairs of chromosomes are shown and different colors stand for different parental origin of each pair of homologous chromosome. Red oval on chromosome 7 represents Th-cre allele whereas paired triangle on chromosome 17 represents floxed Crhr1 allele. Single triangle represents single LoxP site after Cre-mediated excision. In the interphase G1, the primordial cells have one pair of chromosome 7 and one pair of chromosome 17. After the cells enter meiosis I and underwent chromosome duplication, each chromosome has two chromatids and homologous chromosomes pairs, which is followed by crossing over (which is not shown in the diagram for simplicity). After meiosis I, each daughter cell have random combination of chromosome 7 and 17. In another word, some cells may have both Cre and floxed Crhr1 whereas some may have only cre (and wild type Crhr1) and yet some only floxed crhr1 (and wild type Th). If the expression of Cre does not occur before the end of meiosis I, then there is no way for cells that have only floxed Crhr1 (and wild type Th allele) to have excise Crhr1 allele; and only cells that inherit both Cre and floxed Crhr1 could possibly have the floxed Crhr1 excised at a later stage (Scenario B). In contrast, if the expression of Cre occurs before the end of meiosis I, then the excision of the floxed Crhr1 allele could occur before the end of meiosis I. Therefore the presence of excised Crhr1 allele is independent of the Th-cre allele, i.e., even cells without Cre could have excised Crhr1 allele (Scenario A), which is exactly what we have observed. A possible variation to scenario A is that Cre is expressed right before the end of meiosis I and cells that do not inherit Th-cre-bearing chromosome could still obtain Cre protein and excision could occur after meiosis I.
(TIF)

## References

[1] Branda CS, Dymecki SM. Talking about a revolution: The impact of site-specific recombinases on genetic analyses in mice. Dev Cell. 2004;6(1):7–28. Epub 2004/01/16. doi: S153458070300399X [pii]. . 14723844 10.1016/s1534-5807(03)00399-x

[2] Gu H, Marth JD, Orban PC, Mossmann H, Rajewsky K. Deletion of a DNA polymerase beta gene segment in T cells using cell type-specific gene targeting. Science. 1994;265(5168):103–6. Epub 1994/07/01. . 8016642 10.1126/science.8016642

[3] Tsien JZ, Chen DF, Gerber D, Tom C, Mercer EH, Anderson DJ, et al Subregion- and cell type-restricted gene knockout in mouse brain. Cell. 1996;87(7):1317–26. Epub 1996/12/27. doi: S0092-8674(00)81826-7 [pii]. . 8980237 10.1016/s0092-8674(00)81826-7

[4] Evans MJ, Kaufman MH. Establishment in culture of pluripotential cells from mouse embryos. Nature. 1981;292(5819):154–6. Epub 1981/07/09. . 7242681 10.1038/292154a0

[5] Thomas KR, Capecchi MR. Site-directed mutagenesis by gene targeting in mouse embryo-derived stem cells. Cell. 1987;51(3):503–12. Epub 1987/11/06. doi: 0092-8674(87)90646-5 [pii]. . 2822260 10.1016/0092-8674(87)90646-5

[6] Doetschman T, Gregg RG, Maeda N, Hooper ML, Melton DW, Thompson S, et al Targetted correction of a mutant HPRT gene in mouse embryonic stem cells. Nature. 1987;330(6148):576–8. Epub 1987/12/10. 10.1038/330576a0 . 3683574

[7] Geurts AM, Cost GJ, Freyvert Y, Zeitler B, Miller JC, Choi VM, et al Knockout rats via embryo microinjection of zinc-finger nucleases. Science. 2009;325(5939):433. Epub 2009/07/25. doi: 325/5939/433 [pii] 10.1126/science.1172447 19628861 PMC2831805

[8] Cui X, Ji D, Fisher DA, Wu Y, Briner DM, Weinstein EJ. Targeted integration in rat and mouse embryos with zinc-finger nucleases. Nat Biotechnol. 2011;29(1):64–7. Epub 2010/12/15. doi: nbt.1731 [pii] 10.1038/nbt.1731 . 21151125

[9] Miller JC, Tan S, Qiao G, Barlow KA, Wang J, Xia DF, et al A TALE nuclease architecture for efficient genome editing. Nat Biotechnol. 2011;29(2):143–8. Epub 2010/12/24. doi: nbt.1755 [pii] 10.1038/nbt.1755 . 21179091

[10] Tesson L, Usal C, Menoret S, Leung E, Niles BJ, Remy S, et al Knockout rats generated by embryo microinjection of TALENs. Nat Biotechnol. 2011;29(8):695–6. Epub 2011/08/09. doi: nbt.1940 [pii] 10.1038/nbt.1940 . 21822240

[11] Cong L, Ran FA, Cox D, Lin S, Barretto R, Habib N, et al Multiplex genome engineering using CRISPR/Cas systems. Science. 2013;339(6121):819–23. Epub 2013/01/05. doi: science.1231143 [pii] 10.1126/science.1231143 23287718 PMC3795411

[12] Mali P, Yang L, Esvelt KM, Aach J, Guell M, DiCarlo JE, et al RNA-guided human genome engineering via Cas9. Science. 2013;339(6121):823–6. Epub 2013/01/05. doi: science.1232033 [pii] 10.1126/science.1232033 23287722 PMC3712628

[13] Gaj T, Gersbach CA, Barbas CF 3rd. ZFN, TALEN, and CRISPR/Cas-based methods for genome engineering. Trends Biotechnol. 2013;31(7):397–405. Epub 2013/05/15. doi: S0167-7799(13)00087-5 [pii] 10.1016/j.tibtech.2013.04.004 23664777 PMC3694601

[14] Kim H, Kim JS. A guide to genome engineering with programmable nucleases. Nat Rev Genet. 2014;15(5):321–34. Epub 2014/04/03. doi: nrg3686 [pii] 10.1038/nrg3686 . 24690881

[15] Iannaccone PM, Jacob HJ. Rats! Dis Model Mech. 2009;2(5–6):206–10. Epub 2009/05/02. doi: 2/5-6/206 [pii] 10.1242/dmm.002733 19407324 PMC2675817

[16] Li P, Tong C, Mehrian-Shai R, Jia L, Wu N, Yan Y, et al Germline competent embryonic stem cells derived from rat blastocysts. Cell. 2008;135(7):1299–310. Epub 2008/12/27. doi: S0092-8674(08)01567-5 [pii] 10.1016/j.cell.2008.12.006 19109898 PMC2735113

[17] Buehr M, Meek S, Blair K, Yang J, Ure J, Silva J, et al Capture of authentic embryonic stem cells from rat blastocysts. Cell. 2008;135(7):1287–98. Epub 2008/12/27. doi: S0092-8674(08)01568-7 [pii] 10.1016/j.cell.2008.12.007 . 19109897

[18] Tong C, Li P, Wu NL, Yan Y, Ying QL. Production of p53 gene knockout rats by homologous recombination in embryonic stem cells. Nature. 2010;467(7312):211–3. Epub 2010/08/13. doi: nature09368 [pii] 10.1038/nature09368 20703227 PMC2937076

[19] Tong C, Huang G, Ashton C, Li P, Ying QL. Generating gene knockout rats by homologous recombination in embryonic stem cells. Nat Protoc. 2011;6(6):827–44. Epub 2011/06/04. doi: nprot.2011.338 [pii] 10.1038/nprot.2011.338 21637202 PMC3855261

[20] Brown AJ, Fisher DA, Kouranova E, McCoy A, Forbes K, Wu Y, et al Whole-rat conditional gene knockout via genome editing. Nat Methods. 2013;10(7):638–40. Epub 2013/06/12. doi: nmeth.2516 [pii] 10.1038/nmeth.2516 . 23749298

[21] Gall CM, Hendry SH, Seroogy KB, Jones EG, Haycock JW. Evidence for coexistence of GABA and dopamine in neurons of the rat olfactory bulb. J Comp Neurol. 1987;266(3):307–18. Epub 1987/12/15. 10.1002/cne.902660302 . 2891733

[22] Nunes I, Tovmasian LT, Silva RM, Burke RE, Goff SP. Pitx3 is required for development of substantia nigra dopaminergic neurons. Proc Natl Acad Sci U S A. 2003;100(7):4245–50. Epub 2003/03/26. 10.1073/pnas.0230529100 [pii]. 12655058 PMC153078

[23] Madisen L, Mao T, Koch H, Zhuo JM, Berenyi A, Fujisawa S, et al A toolbox of Cre-dependent optogenetic transgenic mice for light-induced activation and silencing. Nat Neurosci. 2012;15(5):793–802. Epub 2012/03/27. doi: nn.3078 [pii] 10.1038/nn.3078 22446880 PMC3337962

[24] Liu Z, Brunskill E, Boyle S, Chen S, Turkoz M, Guo Y, et al Second-generation Notch1 activity-trap mouse line (N1IP::CreHI) provides a more comprehensive map of cells experiencing Notch1 activity. Development. 2015;142(6):1193–202. Epub 2015/03/01. doi: dev.119529 [pii] 10.1242/dev.119529 25725069 PMC4360173

[25] Fujita M, Shimada S, Nishimura T, Uhl GR, Tohyama M. Ontogeny of dopamine transporter mRNA expression in the rat brain. Brain Res Mol Brain Res. 1993;19(3):222–6. Epub 1993/08/01. . 8412565 10.1016/0169-328x(93)90031-j

[26] Cerruti C, Walther DM, Kuhar MJ, Uhl GR. Dopamine transporter mRNA expression is intense in rat midbrain neurons and modest outside midbrain. Brain Res Mol Brain Res. 1993;18(1–2):181–6. Epub 1993/04/01. . 8479287 10.1016/0169-328x(93)90187-t

[27] Nagatsu T. Tyrosine hydroxylase: human isoforms, structure and regulation in physiology and pathology. Essays Biochem. 1995;30:15–35. Epub 1995/01/01. . 8822146

[28] Weber T, Schonig K, Tews B, Bartsch D. Inducible gene manipulations in brain serotonergic neurons of transgenic rats. PLoS ONE. 2011;6(11):e28283 Epub 2011/12/06. 10.1371/journal.pone.0028283 PONE-D-11-14538 [pii]. 22140568 PMC3226688

[29] Witten IB, Steinberg EE, Lee SY, Davidson TJ, Zalocusky KA, Brodsky M, et al Recombinase-driver rat lines: tools, techniques, and optogenetic application to dopamine-mediated reinforcement. Neuron. 2011;72(5):721–33. Epub 2011/12/14. doi: S0896-6273(11)00963-9 [pii] 10.1016/j.neuron.2011.10.028 22153370 PMC3282061

[30] Sato Y, Endo H, Ajiki T, Hakamata Y, Okada T, Murakami T, et al Establishment of Cre/LoxP recombination system in transgenic rats. Biochem Biophys Res Commun. 2004;319(4):1197–202. Epub 2004/06/15. 10.1016/j.bbrc.2004.04.204 S0006291X04008101 [pii]. . 15194493

[31] Ma Y, Ma J, Zhang X, Chen W, Yu L, Lu Y, et al Generation of eGFP and Cre knockin rats by CRISPR/Cas9. FEBS J. 2014;281(17):3779–90. Epub 2014/07/22. 10.1111/febs.12935 . 25039742

[32] Schonig K, Weber T, Frommig A, Wendler L, Pesold B, Djandji D, et al Conditional gene expression systems in the transgenic rat brain. BMC Biol. 2012;10:77. Epub 2012/09/05. doi: 1741-7007-10-77 [pii] 10.1186/1741-7007-10-77 22943311 PMC3520851

[33] Taniguchi H, He M, Wu P, Kim S, Paik R, Sugino K, et al A resource of Cre driver lines for genetic targeting of GABAergic neurons in cerebral cortex. Neuron. 2011;71(6):995–1013. Epub 2011/09/29. doi: S0896-6273(11)00679-9 [pii] 10.1016/j.neuron.2011.07.026 21943598 PMC3779648

[34] Beck IM, Sedlacek R. TT2014 meeting report on the 12th Transgenic Technology meeting in Edinburgh: new era of transgenic technologies with programmable nucleases in the foreground. Transgenic Res. 2015;24(1):179–83. Epub 2014/12/06. doi: 10.1007/s11248-014-9856-2 . 25477276

[35] Vooijs M, Ong CT, Hadland B, Huppert S, Liu Z, Korving J, et al Mapping the consequence of Notch1 proteolysis in vivo with NIP-CRE. Development. 2007;134(3):535–44. Epub 2007/01/12. doi: 134/3/535 [pii] 10.1242/dev.02733 17215306 PMC2583343

[36] Zhang J, Dublin P, Griemsmann S, Klein A, Brehm R, Bedner P, et al Germ-line recombination activity of the widely used hGFAP-Cre and nestin-Cre transgenes. PLoS One. 2013;8(12):e82818 Epub 2013/12/19. 10.1371/journal.pone.0082818 PONE-D-12-09868 [pii]. 24349371 PMC3857304

[37] Winkeler CL, Kladney RD, Maggi LB Jr., Weber JD. Cathepsin K-Cre causes unexpected germline deletion of genes in mice. PLoS One. 2012;7(7):e42005 Epub 2012/08/04. 10.1371/journal.pone.0042005 PONE-D-12-17482 [pii]. 22860046 PMC3409209

[38] Ma Y, Zhang X, Shen B, Lu Y, Chen W, Ma J, et al Generating rats with conditional alleles using CRISPR/Cas9. Cell Res. 2014;24(1):122–5. Epub 2013/12/04. doi: cr2013157 [pii] 10.1038/cr.2013.157 24296780 PMC3879705

[39] Liu Z, Turkoz A, Jackson EN, Corbo JC, Engelbach JA, Garbow JR, et al Notch1 loss of heterozygosity causes vascular tumors and lethal hemorrhage in mice. J Clin Invest. 2011;121(2):800–8. Epub 2011/01/27. doi: 43114 [pii] 10.1172/JCI43114 21266774 PMC3026721

[40] Liu Z, Chen S, Boyle S, Zhu Y, Zhang A, Piwnica-Worms DR, et al The extracellular domain of Notch2 increases its cell-surface abundance and ligand responsiveness during kidney development. Dev Cell. 2013;25(6):585–98. Epub 2013/06/29. doi: S1534-5807(13)00316-X [pii] 10.1016/j.devcel.2013.05.022 23806616 PMC3710456

[41] Satoh A, Brace CS, Ben-Josef G, West T, Wozniak DF, Holtzman DM, et al SIRT1 promotes the central adaptive response to diet restriction through activation of the dorsomedial and lateral nuclei of the hypothalamus. J Neurosci. 2010;30(30):10220–32. Epub 2010/07/30. doi: 30/30/10220 [pii] 10.1523/JNEUROSCI.1385-10.2010 20668205 PMC2922851

