# Correction: Tissue Specific Expression of Cre in Rat Tyrosine Hydroxylase and Dopamine Active Transporter-Positive Neurons

**DOI:** 10.1371/journal.pone.0218976

**Published:** 2019-06-20

**Authors:** Zhenyi Liu, Andrew Brown, Dan Fisher, Yumei Wu, Joe Warren, Xiaoxia Cui

The email address listed for the corresponding author, Xiaoxia Cui, is no longer correct. Dr. Cui’s email address is: x.cui@wustl.edu.

There is an error in Fig 3 of the published article [1]. Specifically, Fig 3 panels B-B" are duplicates of Fig 6 panels B-B", and Fig 3 panel B" is vertically inverted. A revised Fig 3 is provided here.

**Fig 3:**
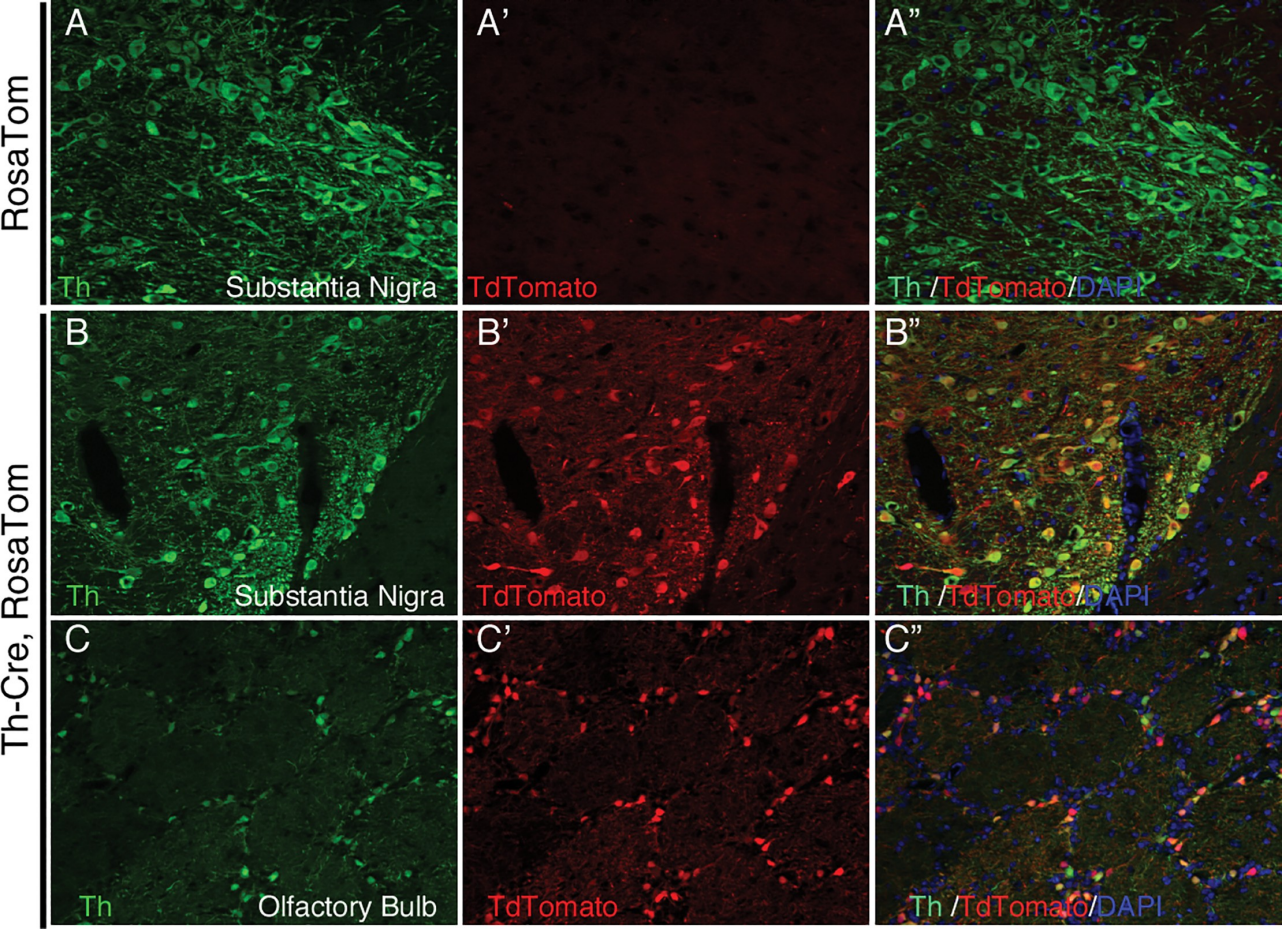
Tdtomato labels Th positive cells in the substantia nigra area of Th-cre, Rosa Tom rat brain. (A-A”), anti-TH antibody staining was done on Rosa Tom brain sections and no live tdTomato signal could be detected; In contrast, in both SN (B-B”) and OB (C-C”), co-localization of TH antibody signal and live tdTomato signals could be observed.

With this Correction, the authors provide the underlying image files for Figs 3, 6 and 7 as Supporting Information S1 File.

## Supporting information

S1 File Supplementary images. This file contains underlying image data for Figs 3, 6 and 7 of the published article [1].
(ZIP)
