# Supplementary figures and images for: Tissue Specific Expression of Cre in Rat Tyrosine Hydroxylase and Dopamine Active Transporter-Positive Neurons
Source: PLoS One. 2016 Feb 17;11(2):e0149379. doi: 10.1371/journal.pone.0149379 (PMC4757100; doi:10.1371/journal.pone.0149379)

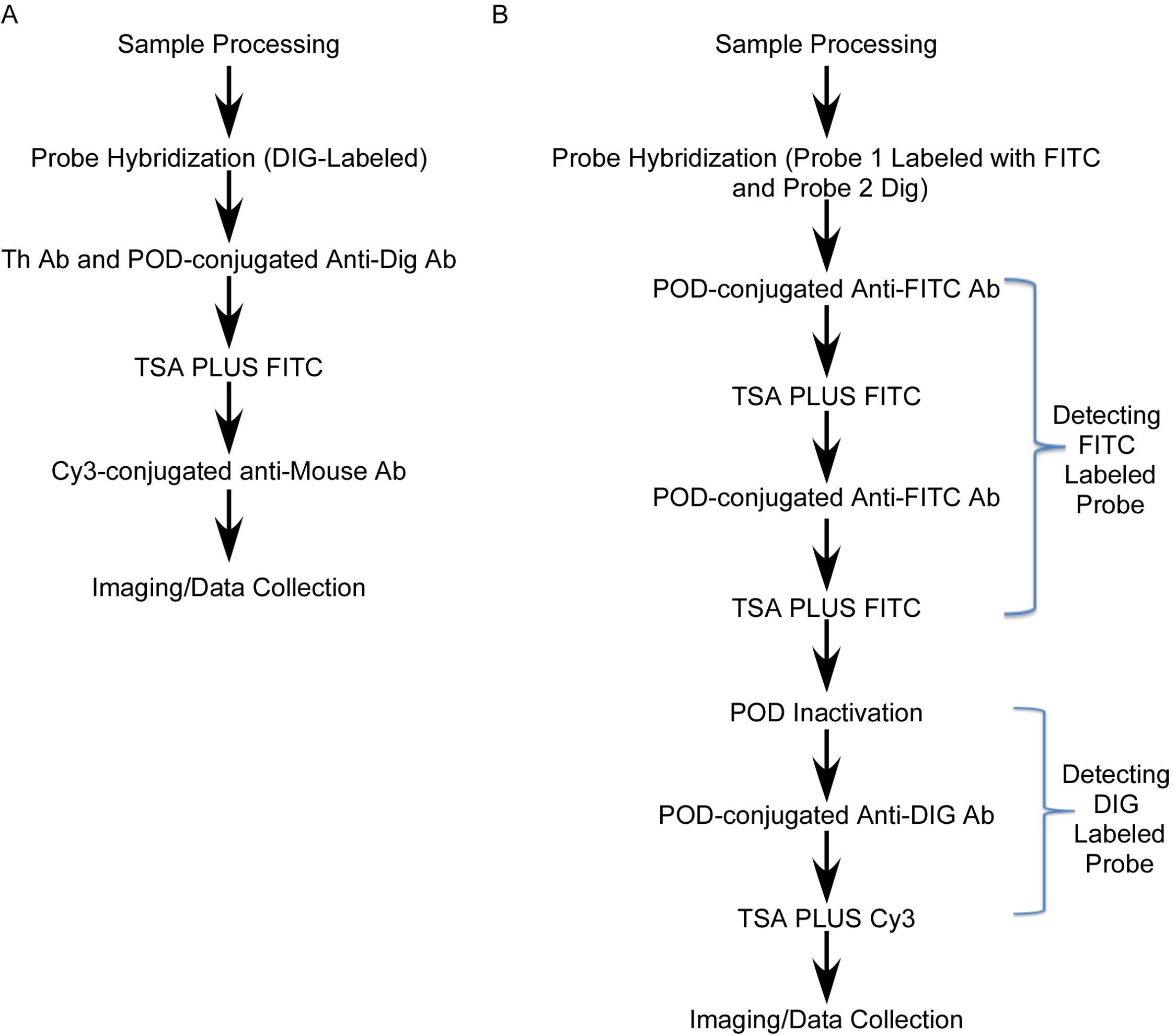

Supplement: S1 Fig — Flow charts for combined of FISH and IHC (A) or double probe FISH (B). Note that two rounds of tyramide signal amplification (TSA) were employed to enhance sensitivity in B. Ab, antibody; Cy3, Cyanine 3; DIG, Digoxigenin; FITC, fluorescein; POD, peroxidase. (TIF) [file pone.0149379.s001.tif]

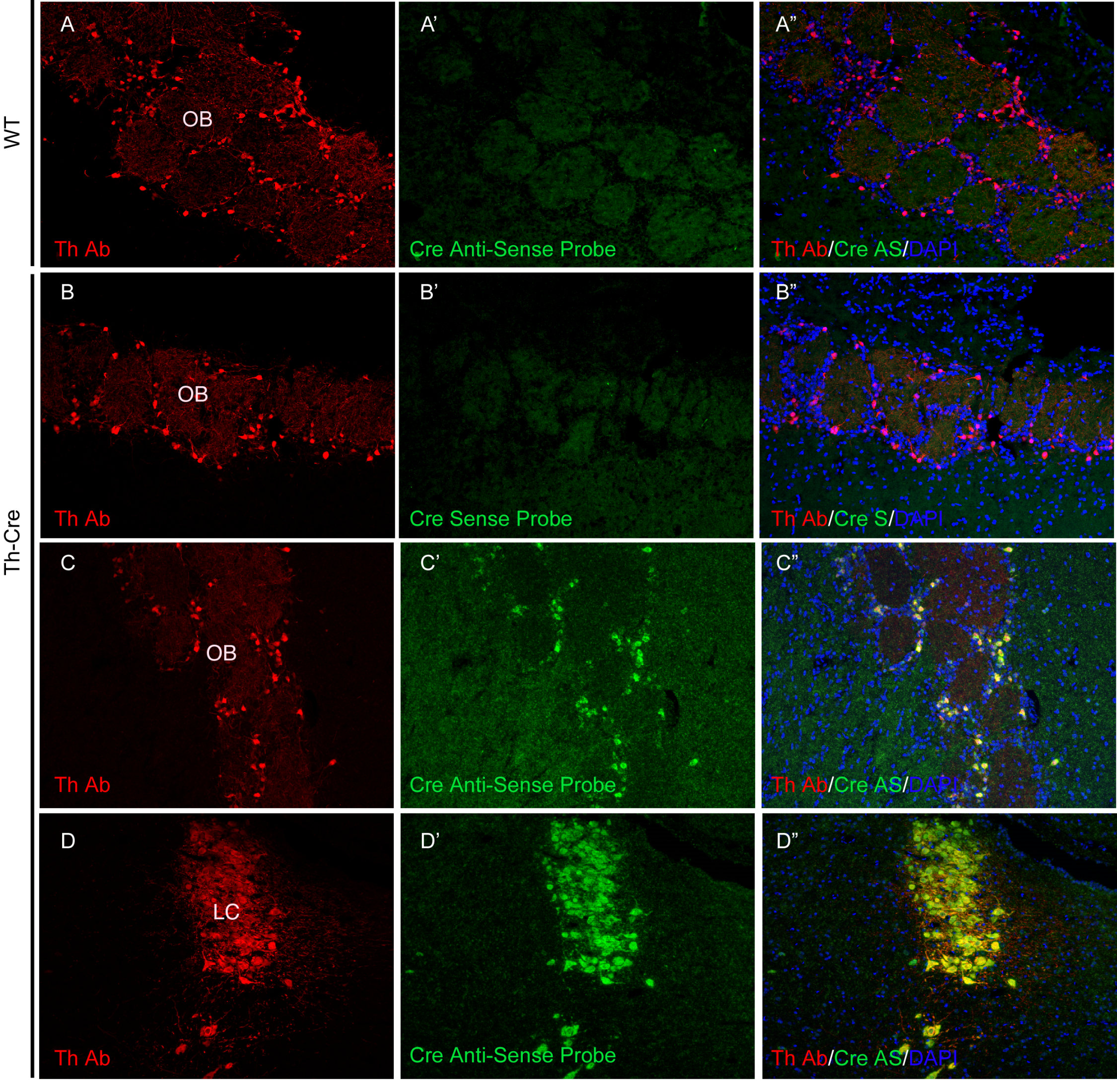

Supplement: S2 Fig — Double staining with Th antibody (red) and cre anti-sense probe (green) revealed their co-localization in the olfactory bulb (C-C”) and locus coeruleus (D-D”). OB, olfactory bulb; LC, locus coeruleus; S probe, sense probe; AS probe, anti-sense probe. (TIF) [file pone.0149379.s002.tif]

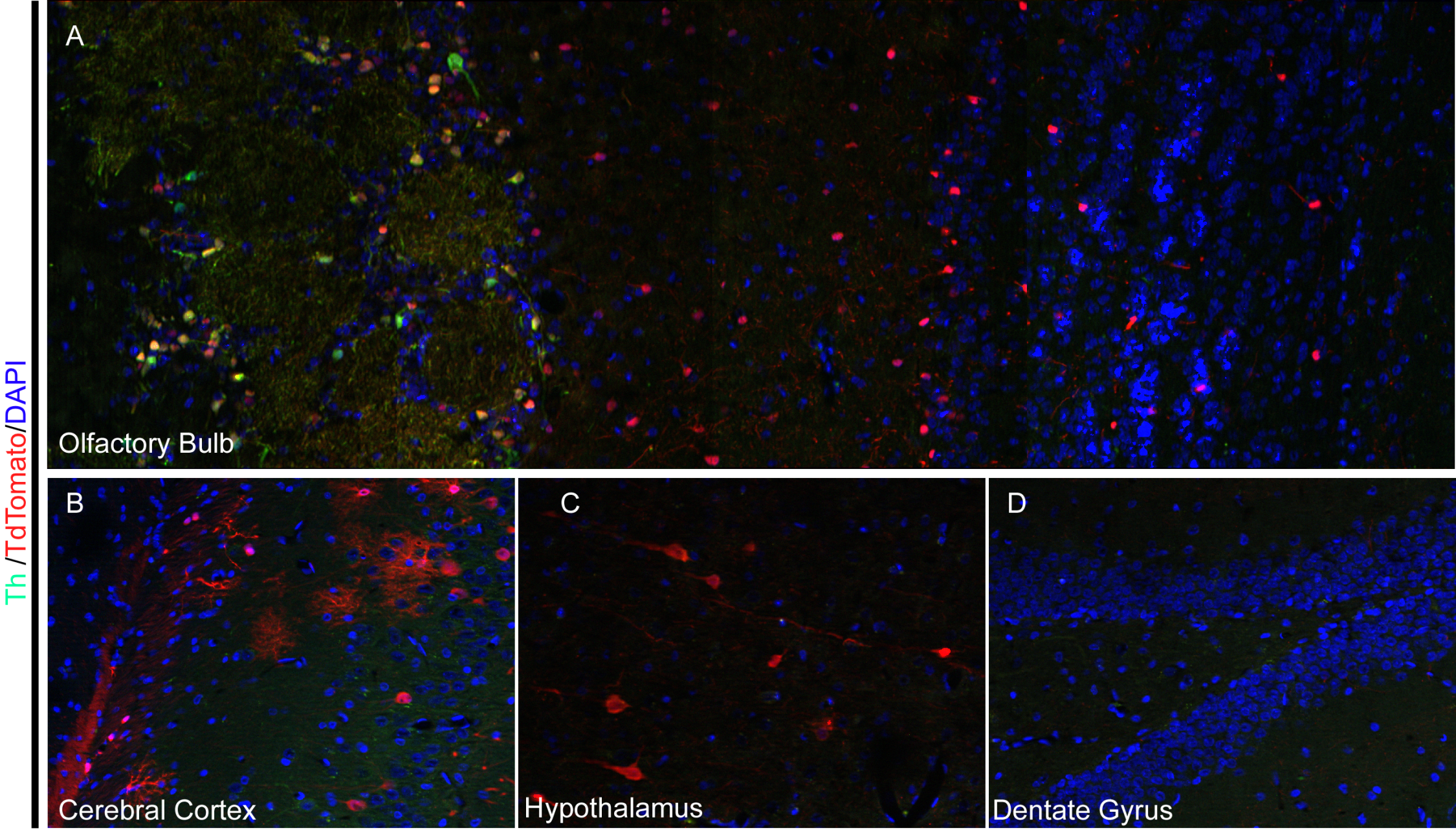

Supplement: S3 Fig — (A) In addition to Th positive neurons, some Th negtive cells in the olfactory bulb are also labeled (Anterior is on the left). A few other Th negative cells were also observed in some other brain areas, including cerebral cortex (B) and hypothalamus (C), but not in the dentate gyrus (D). (TIF) [file pone.0149379.s003.tif]

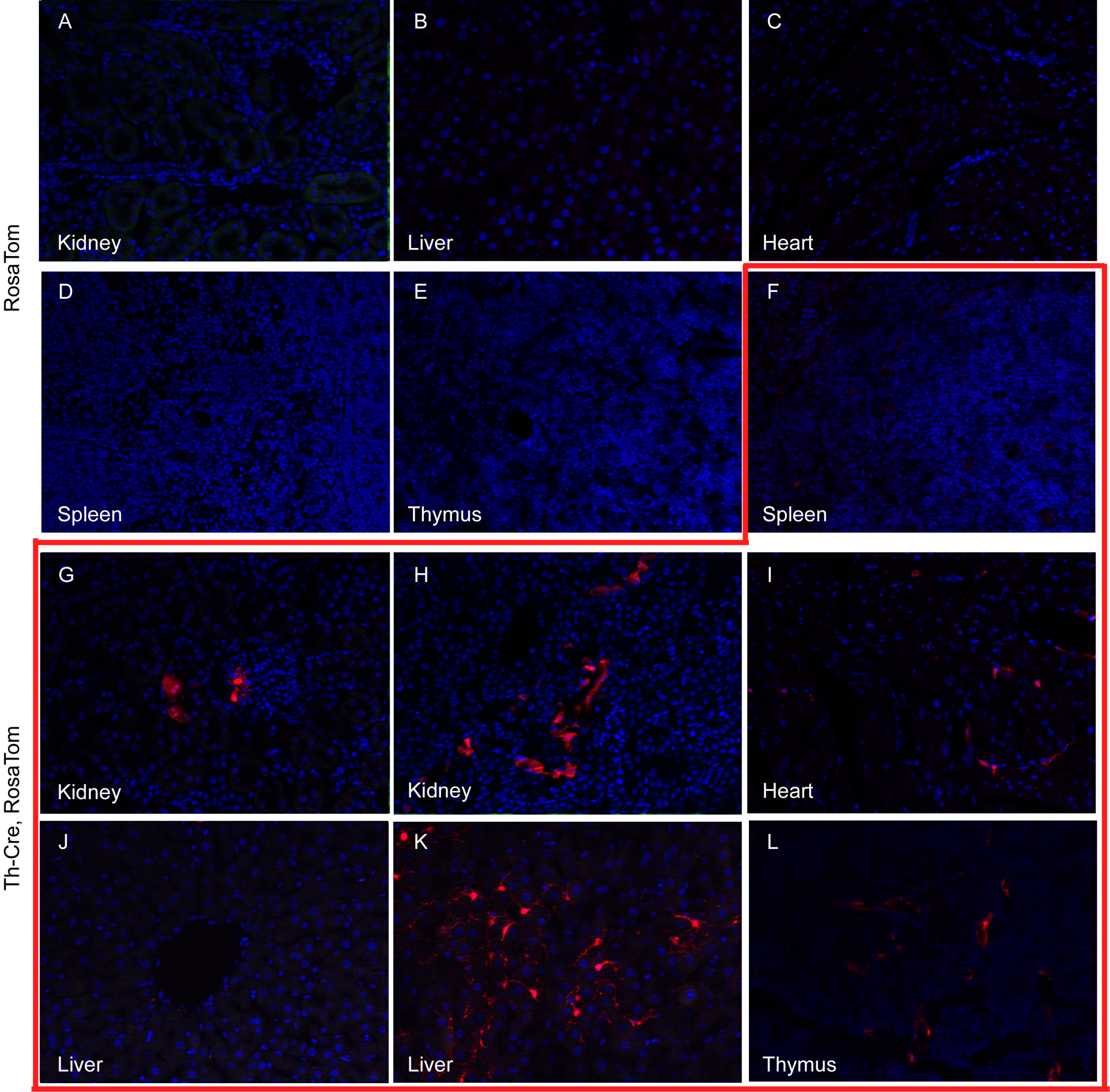

Supplement: S4 Fig — No tdTomato labeled cells were found in any adult organs of RosaTom rat (A-E), confirming that there is no leakage expression of tdTomato reporter in the absence of Cre activity. In contrast, a few tdTomato labeled cells could be observed in some adult organs of Th-Cre, RosaTom rats, including kidney (G and H), heart (I), liver (J and K) and thymus (L). No labeled cells could be observed in the spleen (F). It is unclear whether the labeling reflects ectopic Cre expression or historic transient expression of Th gene. (TIF) [file pone.0149379.s004.tif]

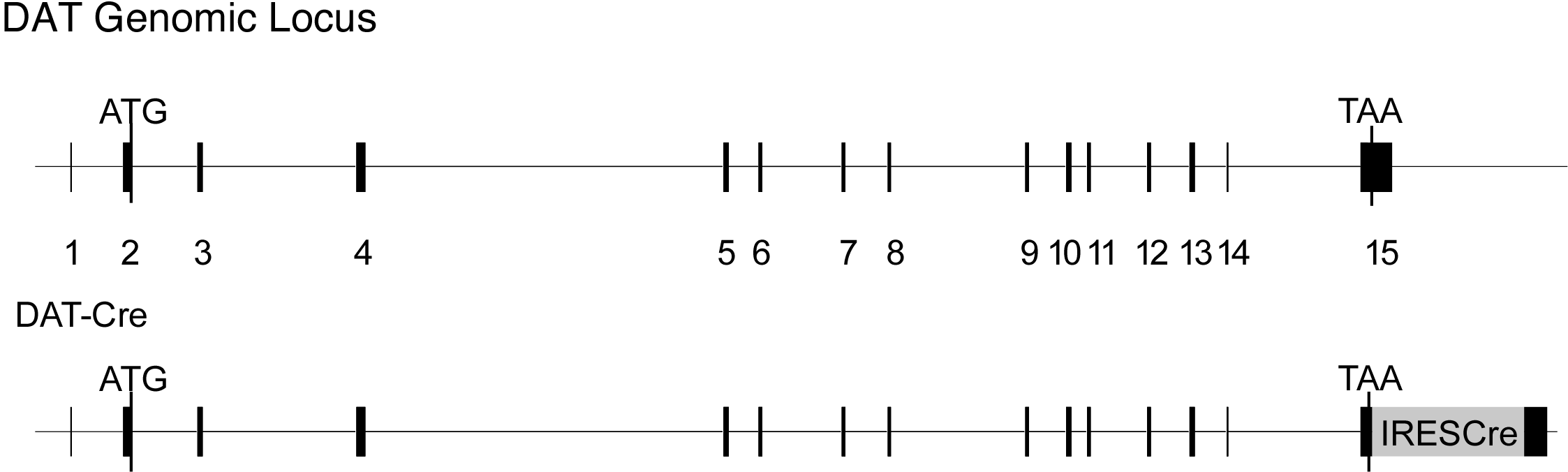

Supplement: S5 Fig — Top panel shows the wild type DAT locus and the bottom panel, with IRES-Cre inserted immediately after the translational stop codon of DAT gene. (TIF) [file pone.0149379.s005.tif]

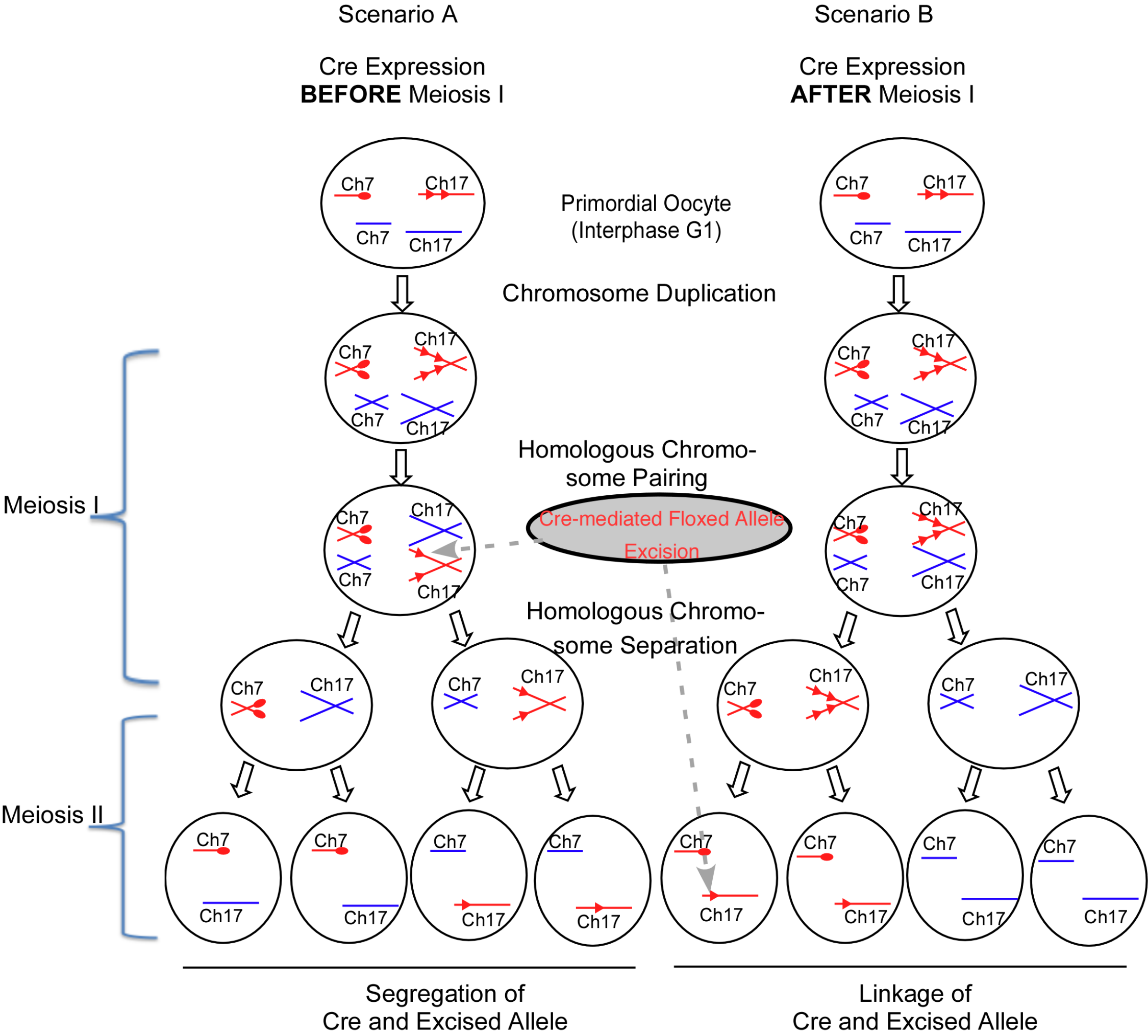

Supplement: S6 Fig — Th-cre is located on chromosome 7 whereas Crhr1 gene is on chromosome 17. And for simplicity, only these two pairs of chromosomes are shown and different colors stand for different parental origin of each pair of homologous chromosome. Red oval on chromosome 7 represents Th-cre allele whereas paired triangle on chromosome 17 represents floxed Crhr1 allele. Single triangle represents single LoxP site after Cre-mediated excision. In the interphase G1, the primordial cells have one pair of chromosome 7 and one pair of chromosome 17. After the cells enter meiosis I and underwent chromosome duplication, each chromosome has two chromatids and homologous chromosomes pairs, which is followed by crossing over (which is not shown in the diagram for simplicity). After meiosis I, each daughter cell have random combination of chromosome 7 and 17. In another word, some cells may have both Cre and floxed Crhr1 whereas some may have only cre (and wild type Crhr1) and yet some only floxed crhr1 (and wild type Th). If the expression of Cre does not occur before the end of meiosis I, then there is no way for cells that have only floxed Crhr1 (and wild type Th allele) to have excise Crhr1 allele; and only cells that inherit both Cre and floxed Crhr1 could possibly have the floxed Crhr1 excised at a later stage (Scenario B). In contrast, if the expression of Cre occurs before the end of meiosis I, then the excision of the floxed Crhr1 allele could occur before the end of meiosis I. Therefore the presence of excised Crhr1 allele is independent of the Th-cre allele, i.e., even cells without Cre could have excised Crhr1 allele (Scenario A), which is exactly what we have observed. A possible variation to scenario A is that Cre is expressed right before the end of meiosis I and cells that do not inherit Th-cre-bearing chromosome could still obtain Cre protein and excision could occur after meiosis I. (TIF) [file pone.0149379.s006.tif]
